# A global review of penalties for abortion-related offences in 182 countries

**DOI:** 10.1136/bmjgh-2022-010405

**Published:** 2023-02-14

**Authors:** Sanhita Ambast, Hazal Atay, Antonella Lavelanet

**Affiliations:** Department of Sexual and Reproductive Health and Research and UNDP-UNFPA-UNICEF-WHO-World Bank Special Programme of Research, Development and Research Training in Human Reproduction (HRP), World Health Organization, Geneve, Switzerland

**Keywords:** Health policy, Maternal health

## Abstract

Public health research and human rights bodies have demonstrated the risks involved with criminalising abortion services and noted a need for full decriminalisation. Despite this, abortions are criminalised in some circumstances in almost all countries in the world today. This paper uses data from the Global Abortion Policies Database (GAPD) to analyse what criminal penalties exist for those who are seeking, providing and assisting in abortions in 182 countries.

This paper uses data on abortion-related penalties available on the GAPD as of October 2022. It includes which actors are penalised, whether specific penalties exist for negligence, non-consensual abortions, whether any secondary additional considerations/judicial discretion exist in sentencing and the legal sources for these penalties.

134 countries penalise abortion-seekers, 181 countries penalise abortion-providers and 159 countries penalise persons assisting in abortions. The maximum penalty is between 0 and 5 years of imprisonment in a majority of countries; however, it can be much higher in other countries. Some countries further prescribe fines, and professional sanctions for providers and those who assist. 34 countries restrict the dissemination of information about abortion.

The range of possible penalties across countries and associated aggravating and mitigating factors for imposing these penalties support arguments for the decriminalisation of abortion on the grounds of arbitrariness. Abortions are also predominantly regulated through the criminal law, which may compound the stigma associated with seeking, assisting with and/or providing abortions when it is criminalised.

There has been no comprehensive study of penalties for abortion at a global level. This article describes what specific penalties abortion seekers and providers face, what factors may increase or decrease these penalties, and the legal sources for these penalties. The findings provide additional evidence of the arbitrariness and potential for stigma associated with the criminalisation of abortion and strengthen the case for decriminalisation.

**Summary Box**

- While public health research and human rights bodies have demonstrated the risks involved with criminalizing abortion services and noted a need for their full decriminalization, there has been no comprehensive study of penalties for abortion at a global level.
- This article describes what specific penalties abortion seekers and providers face, what factors may increase or decrease these penalties, and the legal sources for these penalties.
- The findings provide additional evidence of the arbitrariness and potential for stigma associated with the criminalization of abortion and strengthen the case for decriminalization.

## Introduction

Public health research and human rights bodies have demonstrated the risks involved with criminalising abortion services and noted a need for their full decriminalisation.^1–4^ Evidence indicates that criminalisation does not impact the decision to have an abortion, prevent women from having abortions or prevent women from seeking information regarding where they can access abortions.^2–4^ Rather, criminalisation limits or delays access to safe abortion and increases the possibility of women and girls resorting to unsafe and unregulated abortion services.^5^ It imposes a range of burdens on women including unnecessary travel and cost, delayed or no access to postabortion care, distress and stigma.^6–8^ It can also discourage people from seeking postabortion care and accessing safe abortion services when they are available.

Criminalisation of abortion may also lead to people being punished in other circumstances, such as miscarriages. Often, these burdens fall more heavily on women and girls who experience other forms of marginalisation, including poverty.^3^ Criminalisation can cause health workers to act cautiously, even where abortion is legal,^1 9^ and can also contribute to misdocumentation or refusal to provide care. It also contributes to the lower availability of trained abortion providers and a loss of relevant skills in the health workforce.^8^ Recognising a range of human rights violations, including gender-based discrimination and violence; torture and/or ill treatment; as well as violations of the rights to life, health and privacy, United Nation (UN) treaty bodies and special procedures have called for the decriminalisation of abortion.^10–14^

Appreciating the impacts on health and human rights protection and enjoyment, the WHO recommends the full decriminalisation of abortion.^8^ Decriminalisation means ‘removing abortion from all penal/criminal laws, not applying other criminal offences (eg, murder, manslaughter) to abortion and ensuring there are no criminal penalties for having, assisting with, providing information about, or providing abortion, for all relevant actors’.^8^ The Abortion Care Guideline makes clear that while decriminalisation is a necessary step for the legalisation of abortion, ensuring that quality abortion is available and accessible may require further legal or regulatory changes beyond decriminalisation.

Despite the concerns associated with criminalisation, abortions are criminalised in some circumstances in almost all countries in the world today.^15^ In 11 countries, abortion is completely criminalised. In one country, Canada, abortions carry no criminal penalties for any circumstances. Abortion is usually available on certain grounds or until a specified gestational age (linked to particular grounds).^16^ Outside of these circumstances, it is considered a criminal offence. Where abortion is lawfully available, it is commonly regulated through both the criminal law and healthcare law, unlike other health services.^8^Further compounding this issue is that federal countries also regulate abortion in varied ways across subnational jurisdictions. For example, in Mexico, while several states have decriminalised abortion before 13 weeks, others have not.^17^ Similarly, regulation of abortion now happens at the state level in the USA after a recent Supreme Court decision, and many states have restricted access and increased penalties.^18^

### Data collection

This paper uses data from the Global Abortion Policies Database (GAPD) to better understand what criminal penalties exist for actions associated with seeking and providing abortions globally. The GAPD aims to increase transparency of information and accountability of states for the protection of individuals’ health and human rights. It contains information related to abortion regulation and service delivery for all WHO member states. The methodology for how the GAPD is coded is explained in detail elsewhere.^19^ The GAPD presents the source documents for penalties for abortion care; it does not offer information related to the meaning of legal texts or how legal texts are interpreted. It uses unofficial translations where necessary.^15^

In this paper, criminalisation of abortion refers to the regulation of behaviours associated with abortion seeking and provision through the criminal law. ‘Penalties’ in this paper refer to criminal penalties.

The GAPD documents the legal provisions that criminalise abortions, including the specific penalties for the person seeking an abortion, providers and people assisting with an abortion. A total of 182 countries have been included in this analysis. No data on offences and penalties for abortion could be found for four countries (Marshall Islands, Micronesia, North Korea and Eswatini); these have been excluded from this analysis.

Nine countries have been excluded because the regulation of abortion varies within subnational jurisdictions in the country (Nigeria, Bosnia, UK, Mexico, USA, Australia, China, Switzerland and Canada). While discussing the range of possible penalties for abortion-related offences, we categorise countries by the maximum prison term possible for abortions conducted with the consent of the person seeking them. Specifically, countries have been organised by those which have life imprisonment; a maximum prison term of above 10 years; between 5 and 10 years; and 5 years or less. Where additional aggravating factors lead to higher prison sentences, this is specifically mentioned. Where penalties consist of fines, we have not analysed or compared the amount of the fine applicable, given variations in currency and context. Where the number of countries in any category of analysis is under 10, we have listed them all in text or in the references.

We used a standardised extraction form to collect information about all abortion-related offences including type of penalty and time frames; and aggravating or mitigating factors. One author extracted the data (SA); a second author cross-checked the data (HA). Any discrepancies were reviewed and discussed with a third reviewer (AL).

We analyse information reflected in the GAPD and, are thus, reliant on the methodology employed by the GAPD. Countries are grouped by UN regional groups: Africa, Asia, Europe, Oceania, Latin America and North America. North America, however, contains only Canada and USA of which the USA is excluded from this paper, and Canada has no penalties for abortion. Therefore, the graphs in this paper do not have any data from North America.

This paper uses data available on the GAPD as of October 2022. The GAPD is updated at the point at which a new source becomes known or available, which means there may be reform not currently reflected. We are although limited by the fact that the GAPD provides access to country sources, but does not include information about how these laws or policies are implemented on the ground. Yet, by reflecting how abortion is regulated through criminal law, we seek to provide more specific information about how abortion is regulated across countries, and highlight any patterns in this regulation.

## Review of what penalties different actors involved in an abortion face

### General overview of abortion criminalisation

In 163 countries, the definition of, and penalties for, abortion-related offences are contained in the general penal code. In 12 countries, the offences and penalties for abortion are found in abortion-specific laws. In eight countries, they are found in other types of legal sources, such as health codes, reproductive health laws and laws about children (Benin, Burkina Faso, Madagascar, Mali, Mauritania, France, Saudi Arabia and Denmark). Many of the 163 countries where abortion-related offences are found in the general penal code also have abortion-specific and health care-specific laws.^15^ However, these sources do not prescribe the offences and penalties for abortion.

In most countries, abortions are criminalised in some circumstances. In 11 countries, abortion is completely criminalised and prohibited in all circumstances.

In countries where abortions are criminalised, a range of actors are commonly subject to penalties. In 134 countries, the person seeking the abortion is penalised. Providers of abortion services are subject to criminal penalties in 181 countries. A total of 159 countries penalise persons who assist in accessing or providing abortions. While some countries penalise the persons seeking an abortion with a higher penalty, in other countries, the provider is potentially subject to more stringent punishments. In almost all countries, the person assisting could receive the same, or lower penalty, than the provider.

**Figure 1:**
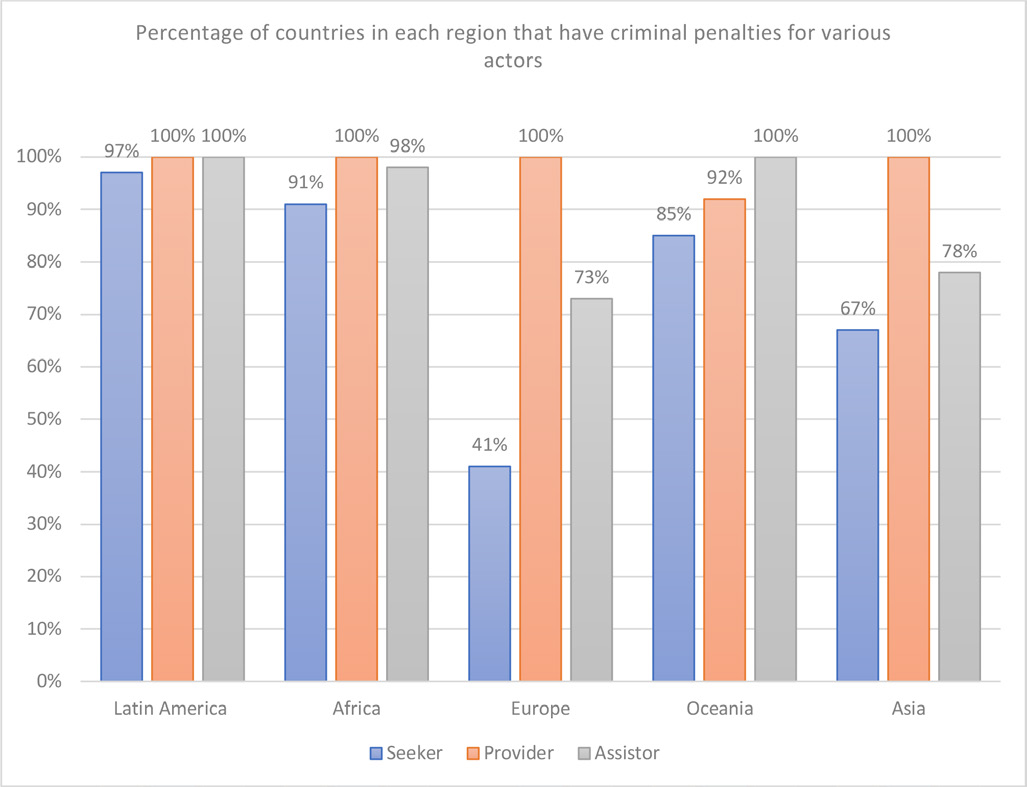
Percentage of countries in each region that have criminal penalties for various actors.

Figure 1 provides detail by region about which actors are criminalised.

Other actors are specifically mentioned in some countries, examples include: anyone who ‘knowingly makes a false declaration of rape, sexual intercourse with a female under 16 or sexual intercourse with a specified person’ to the police ‘for the purpose of procuring treatment to terminate a pregnancy’ (Mauritius); the parents of the person getting an abortion may be penalised (Philippines); and the managers of health institutions in which an unlawful abortion has taken place (France).

There is a wide range of penalties that people convicted of abortion-related offences can face across countries. Imprisonment and fines are the most common. The type of penalty varies depending on the actor being penalised.

The actors and actions penalised are almost identical among some groups of countries. For example, five countries (Jamaica, Barbados, Trinidad and Tobago, Sierra Leone and St Kitts and Nevis) have legislation called the ‘Offences against Persons Act’, and the text of the provisions criminalising abortions is very similar to each other.^20^ In 21 different countries, abortion is criminalised using a provision that is similar to the following text: ‘whoever, by food, drinks, drugs, violence or by any other means, procures abortion of a pregnant woman or whether she has consented or not, shall be punished by imprisonment’.

### Person seeking an abortion

In 134 countries, where the individual seeking an abortion can be penalised, this is done through different types of provisions. Many countries penalise ‘a woman with child’ who ‘unlawfully’ ingests a ‘drug’ or ‘poison or other noxious thing’ or ‘uses any instrument’ to ‘procure her own miscarriage’. In some countries, the crime of abortion is defined (eg, ‘intentionally and unlawfully causing abortion or miscarriage’), and the person seeking an abortion is mentioned as someone who can be penalised under it.

In almost all countries where the person seeking an abortion is criminalised, imprisonment is a possible penalty. Laws usually prescribe a range of possible prison time that may be imposed based on the judge’s discretion. In 91 countries, the maximum penalty is between 0 and 5 years of imprisonment for a consensual abortion, where no aggravating factors apply. In 25 countries, the maximum penalty is between 5 and 10 years, and in 2 countries (Equatorial Guinea, Zambia), the penalty is between 10 years and life imprisonment. In six countries, a person seeking an abortion can be imprisoned for life (Kiribati, Solomon Islands, Tuvalu, Barbados, Belize, Jamaica). In three countries (Dominican Republic, Chile, Haiti), imprisonment for the person seeking an abortion is possible, but maximum terms are not clarified in the law, so they are not reflected in the numbers above.

Forty-eight countries allow for people seeking abortions to be fined. While in some countries, fines can be imposed as an alternative to imprisonment, in most countries, fines can be imposed in addition to a prison sentence.

### Providers

Provisions criminalising providers vary as well. Some countries criminalise whoever, intending to cause an abortion, ‘administers’ or ‘causes to be taken’ any ‘drug’, or ‘poison’ or ‘noxious thing’ or ‘uses any instrument’ for this purpose. Others criminalise any person who ‘interrupts a pregnancy’, or ‘performs’ or ‘procures’ or ‘intentionally causes’ an abortion in prohibited circumstances.

Among the 181 countries that criminalise providers, in 126 countries, the maximum penalty is between 0 and 5 years of imprisonment for an abortion with the person’s consent. In 25 countries, the maximum penalty is between 5 and 10 years, and in 14 countries, the penalty is between 10 years and life imprisonment. In five countries, a provider can be imprisoned for life (Solomon Islands, Barbados, Jamaica, Guyana and Belize). In six countries, the length of the prison term is not clear (Dominican Republic, Chile, Haiti, Belarus, Latvia and Lesotho).

Where aggravating factors apply, the maximum term of imprisonment for the provider can be 20 years or over in nine countries (Algeria, Burundi, Malaysia, Mali, Morocco, Sri Lanka, Thailand, Ivory Coast and Turkey), and life imprisonment in six countries (Benin, Burkina Faso, Rwanda, Singapore, South Sudan and India).

Seventy-six countries prescribe fines for providers of abortions. Forty-eight countries prescribe some form of professional sanction for providers, which include: seizure or forfeiture of equipment, demotion, closure of establishments, official warnings, termination from employment, suspension from practising their profession for a defined period, suspension of qualifications and a complete prohibition from working in the field again, or a ban on holding certain posts.

### Person assisting with an abortion

Individuals who assist in abortions may be penalised in countries where the law specifically criminalises certain ‘assisting’ functions (such as financing abortions or selling equipment that could be used to perform an abortion), or where the law has a broad understanding of what it means to provide an abortion. For example, countries with provisions that penalise anyone who ‘causes’ an abortion, or ‘engages’ in an abortion can potentially apply to both providers and those who assist them in any way.

In 127 countries, the maximum penalty is between 0 and 5 years of imprisonment for people who assist in a consensual abortion, without the application of any aggravating factors. In 16 countries, the maximum penalty is between 5 and 10 years, and in 5 countries, the penalty is between 10 years and life imprisonment (Benin, Democratic Republic of Congo, Ireland, Equatorial Guinea, and Saint Vincent and the Grenadines). In one country (Barbados), a person assisting in an abortion can be imprisoned for life. In four countries, the length of the prison term is not clear (Dominican Republic, Chile, Haiti and Lesotho).

Fifty-nine countries prescribe fines for people who assist in abortions. Thirty-three countries prescribe some form of professional sanction for individuals acting in a medical capacity for assisting in abortions, such as pharmacists who prescribe medicines, and nurses who provide counselling. The nature of professional sanctions faced by people who assist in abortions is similar to those faced by providers of abortions (listed above).

**Figure 2:**
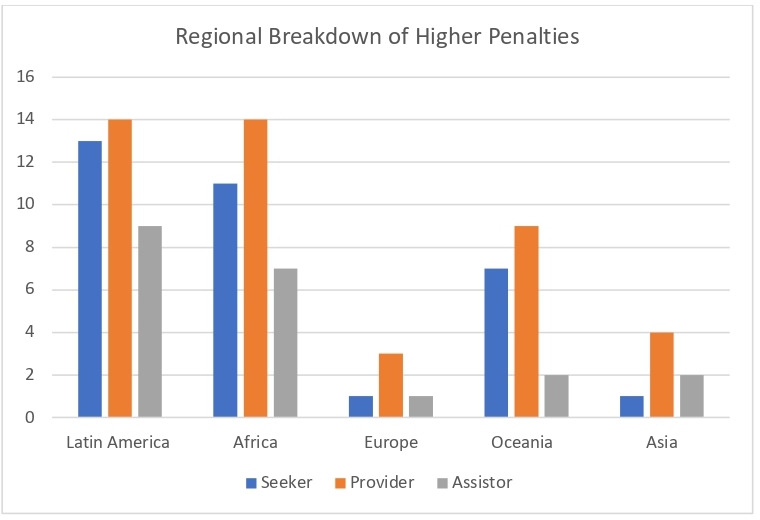
Number of countries in each region with maximum penalties over 5 years.

Figure 2 represents the regional distribution of countries with maximum penalties over 5 years for abortions seekers, providers and those who assist them.

### Other penalties

Some countries also prescribe other penalties not included in the sections above. Penal codes do not always clearly define what each of these penalties entail, and therefore, we have listed them as stated in the text of the law here. These include prohibitions on residence (eg, Mali, Algeria, Mauritania and Morocco),^21^ prohibitions on the exercise of ‘civic and family rights’ (eg, Burkina Faso),^22^ transportation for life (eg, Myanmar),^23^ payment of ‘diya’ or ‘qisas’ (eg, Iran, Pakistan),^24^ payment of ‘blood money’ (Yemen),^25^ ta'zir (eg, Pakistan),^26^ corrective labour (eg, Armenia, Azerbaijan, Kazakhstan, Russia, Tajikistan, Turkmenistan, Uzbekistan, Ukraine), hard labour (eg, Syria, Antigua), compulsory labour (Russia), community service (Georgia, Latvia, Lithuania, Ukraine) and non-custodial reform (Vietnam). Some legal systems also specify variations on imprisonment for abortion-related offences, such as imprisonment with work (Japan) and temporary reclusion (Philippines).

Twenty-four countries prescribe some of these penalties for providers, 15 countries prescribe such penalties for those who assist in abortions and 13 prescribe them for people who seek an abortion.

**Figure 3:**
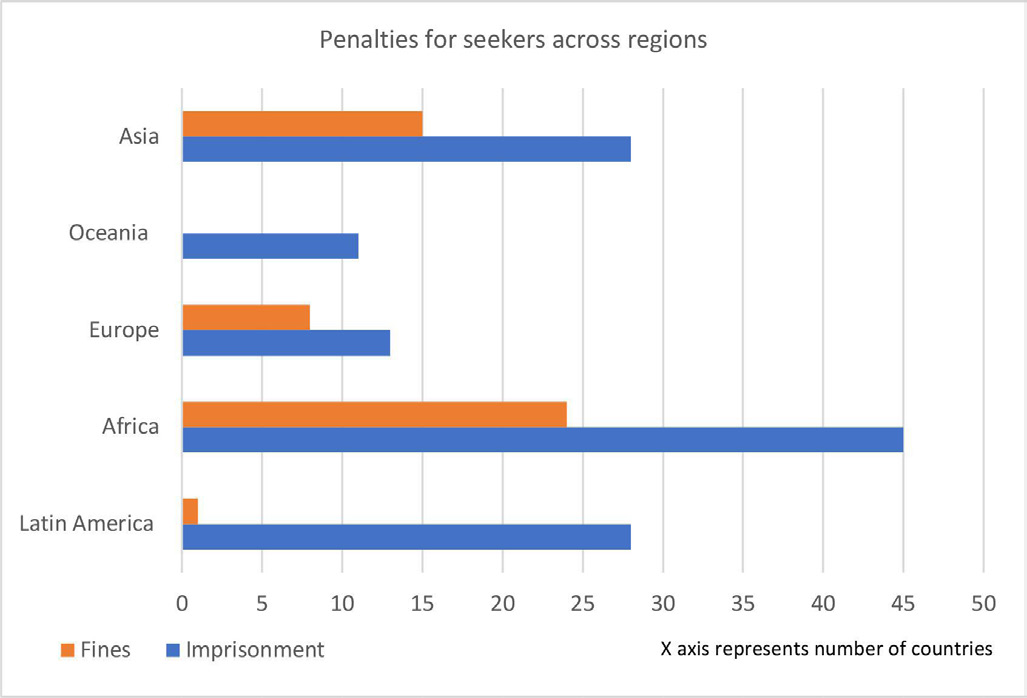
Types of penalties for abortion seekers across regions.

**Figure 4:**
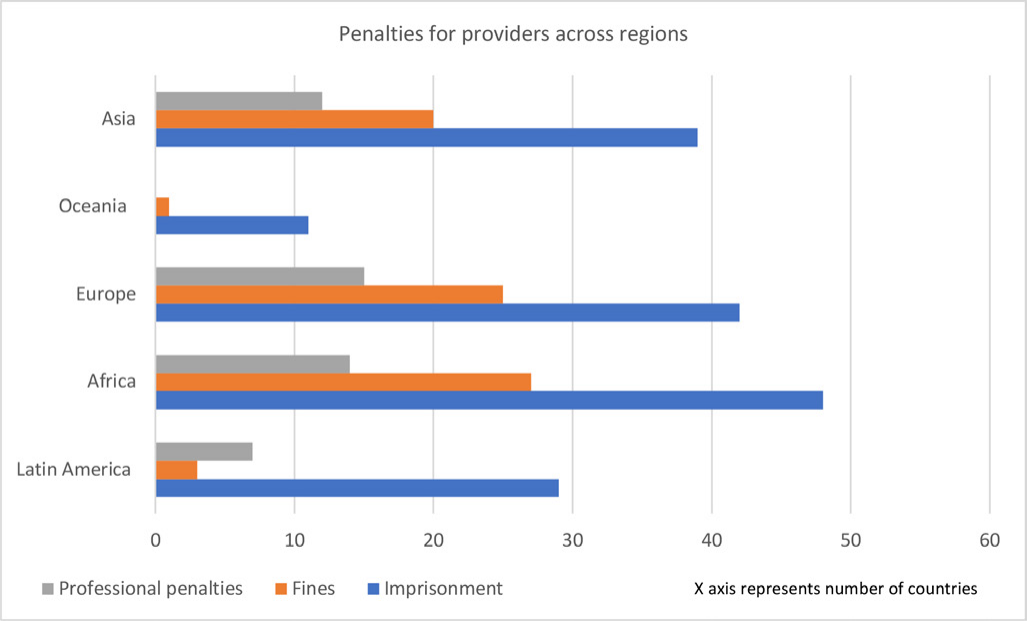
Types of penalties for abortion providers across regions.

**Figure 5:**
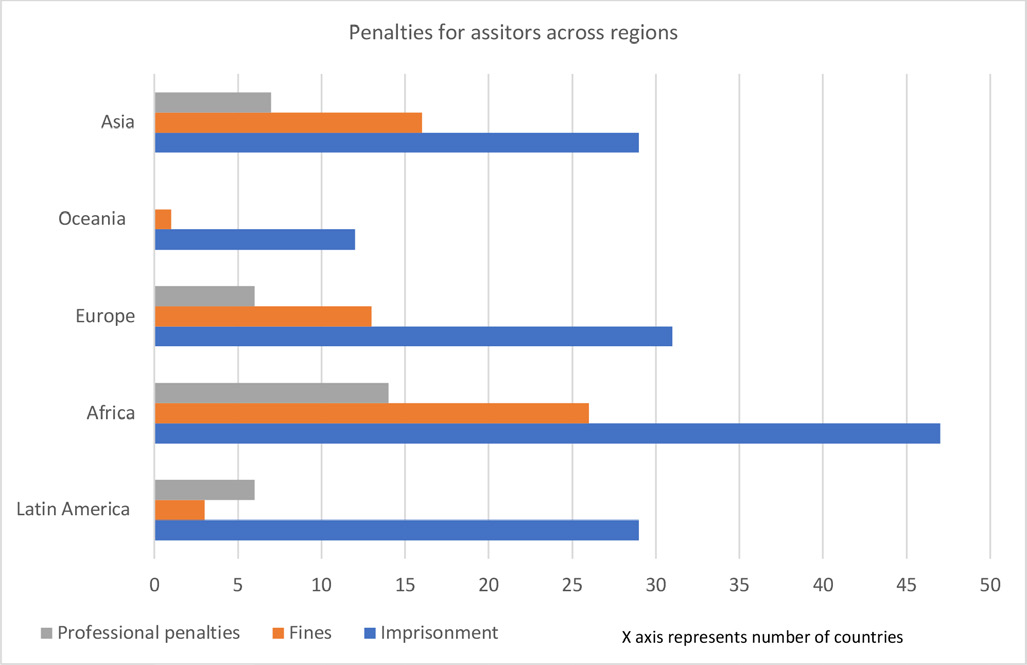
Types of penalties for people who assist in abortions across regions.

Figures 3–5 illustrate the existing types of penalties for abortion seekers, providers and assistors, across regions.

### Aggravating factors for sentencing

In a large majority of countries, the laws specify circumstances that aggravate or increase the applicable penalties in abortion-related offences. In some cases, these factors can be considered at the judge’s discretion, but in most cases, the existence of these factors automatically makes the applicable penalty higher. These factors apply to different types of penalties. Therefore, they may result in higher fines, increased prison sentences and longer professional disqualification, depending on the country in question.

For example, penalties in 76 countries can also be increased if the abortion resulted in the woman or girl’s death or resulted in serious injury. Thirty-three countries prescribe more stringent penalties for providers and those assisting them when they are habitual or repeat offenders. In 13 countries, penalties may be increased if the pregnancy is considered more advanced. This is defined in various ways: in some countries, it is linked to gestational age, while in others it depends on when fetal movement is felt or viability. Other factors that give reason for higher penalties include: if someone has acted for profit or personal gain; if the girl is a minor; if the offender is related to the woman or girl; if certain legal requirements for abortion provision are not met; and if the provider is not a qualified professional.

### Forced or coerced abortions

One common circumstance when the penalty for providing an abortion is increased is when the abortion is carried out without the consent of the woman or girl, or when it is coerced in some manner. Eighty countries contain specific, and higher, penalties for non-consensual abortions as compared with consensual abortions. Thirteen countries, similarly, have aggravated penalties for abortions conducted violently, or with intimidation or deceit.

### Mitigating factors for sentencing

Several penal codes also list factors which can result in the sentence for abortion-related offences being mitigated or reduced. Like with aggravating factors, in some countries, these are framed as circumstances that a judge may refer to at their discretion to reduce a sentence. In others, the provision mandates that the existence of these factors automatically reduces the sentence by a particular amount.

Twenty-seven countries list specific factors that judges can or must refer to, to mitigate penalties in abortion-related offences. One set of mitigating factors involves the consideration of circumstances that amount to legal grounds for abortion in other countries. Examples include where sentences can be reduced when it is shown that the pregnancy was terminated because of risk to physical or mental health (Eritrea, Guatemala, Uruguay), rape (Eritrea, Colombia, Peru, Uruguay), incest (Eritrea), fetal impairment (Peru) and poverty (Eritrea, Ethiopia, Uruguay). Another common mitigating factor is when the pregnancy is terminated to reduce apparent social disapproval or preserve the woman or girl’s ‘honour’. Penalties can also be mitigated in some countries if the provider is related to the woman/girl (Iraq), if the abortion was carried out unintentionally (Afghanistan, Peru, Malta), and if it was carried out because of lack of support for the child (Paraguay). Finally, some countries have an open-ended clause which states that the judge may mitigate a sentence where they see fit (Sao Tome and Principe, Argentina, Colombia, Iceland). For example, the law in Iceland states: ‘… In case of especially extensive mitigating circumstances it may be decided that [the] penalty be cancelled.’^27^ The law in Sao Tome and Principe states, ‘If the abortion provided for in paragraphs 2 and 3 is practised to prevent the woman’s social disapproval, or for reasons that significantly reduce the guilt of the perpetrator, the applicable sentence may not exceed 1 year’.^28^

### Restrictions on the dissemination of information

Penalties for the dissemination of information are also found in different types of legal instruments. In 24 countries, the offences and penalties are contained in the general penal code. In 13 countries (this includes some countries where the penal code also has restrictions on information), it is found in a different legal instrument which includes public health laws, laws on advertising and laws regulating poisons.

Thirty-four countries restrict the dissemination of information about abortion and abortion services, even when abortions may be legal in some circumstances. A range of actions are prohibited under these provisions including: making speeches in public places; advertising medical facilities and services; advertising of ‘procedures, means or objects suitable for termination of pregnancy’; promoting, recommending, exhibiting, publishing, selling or offer to sell items that cause abortions; ‘indicat(ing), favour(ing) or practice(ing) the means of procuring abortion’; any advocacy of ‘the use of any means of aborting a woman’; and ‘Preparing, displaying, selling or in any way being connected with materials that would induce abortions’. Nine of these countries have some exceptions; for example, information provided by doctors to lawfully terminate a pregnancy or information published in scientific journals, is exempt from punishment (Angola, Gabon, Cyprus, Albania, Germany, Greece, Kiribati, Tuvalu, Solomon Islands).

The penalties for the dissemination of information about abortion are generally lower than the penalties that people seeking abortions, providers and those who assist may face.

## Arbitrariness in abortion penalties

As the findings from this paper indicate, a person seeking an abortion faces criminal penalties in 134 countries: they may face a fine in one country and life in prison in another, for the same behaviour. It is the same for providers of abortions and those who assist them. The sheer range of penalties that persons involved in the abortion may face, depending on where they are, support the argument that provisions criminalising abortions are arbitrary. A law is considered arbitrary if it inflicts harm without need or reason, or if its prohibitions bear no connection to or undermine its aims, however legitimate’.^5^ This arbitrariness is also evident in the listed mitigating and aggravating factors that influence sentencing in abortion-related offences. Where sentences are increased because the abortion was coerced or non-consensual, it would not be arbitrary, since these constitute serious assaults. However, as the results demonstrate, the same circumstance, such as if the provider is related to the person seeking the abortion, may be a reason to increase the penalty in one country and decrease the penalty in another.

With regard to abortion, there is evidence that criminalisation does not decrease abortions or make them safer.^3 8^ Therefore, scholars have argued, and international human rights standards have affirmed, that absolute criminal prohibitions on abortion constitute an arbitrary deprivation of the rights to life and health, and hence, have recommended for decriminalisation.^29^ The WHO’s Abortion Care Guideline also notes that criminalisation of abortion delays access to abortion; imposes burdens on abortion seekers including unnecessary travel and cost, delayed or no access to postabortion care, distress and stigma; increases recourse to unlawful and unsafe abortion; contributes to the lower availability of trained abortion providers; and can cause health workers to act cautiously, making them hesitant to provide abortion care in circumstances where it is legal.^8^ Studies in countries where abortion has been fully or partially decriminalised have also noted several benefits to abortion seekers as a result, including access to better quality care,^30^ lower rates of maternal mortality,^31^ and increased educational attainment, career outcomes and earnings.^32^

## Reinforcement of stigma

The results demonstrate that in a vast majority of countries (163) abortions are regulated through the same legal instrument as applies to other offences. Many countries where penalties for abortions exist also have health-related guidelines and regulations.^15^ Notwithstanding this, in many of these countries, in addition to prescribing penalties, the criminal law also serves as the primary source of abortion regulation, including where abortions can be carried out, by whom and when conscientious objection is possible. While this paper has not dealt with the extent and impact of this broader criminal regulation of abortion, this is contrary to public health advice. For example, the WHO’s Abortion Care Guideline recommends that instead of criminal law, abortion should be ‘regulated similarly to other healthcare interventions, that is, by general healthcare law and policy, best practice, training and evidence-based guidelines’. Regulating abortions through the same legal instrument, and same institutional apparatus as murder, sexual assault and robbery may exacerbate the concerns associated with seeking and providing abortions when it is criminalised: it may compound the stigma abortion seekers and providers experience, create a ‘chilling effect’, making people more reluctant to seek and provider abortions, and create barriers to accessing safe abortion services even when legal.^5^

The Abortion Care Guideline further notes that decriminalisation of abortion does not make women, girls or other pregnant persons vulnerable to forced or coerced abortion; these would constitute serious assaults as these would be non-consensual interventions, and covered by general criminal law prohibitions against ‘assault’, ‘grievous hurt’, etc.^8^ And yet, these types of assaults are often expressly criminalised, further exceptionalising the way in which abortion is regulated through the criminal code, and not as healthcare.

Furthermore, some factors that may increase or decrease sentencing risk creating categories of abortion-seekers and providers that are seen as less or more ‘deserving’ of punishment, often based on gender stereotypes, which can further perpetuate stigma. For example, some legal systems give abortion seekers and their family lighter sentences when abortions are sought to ‘preserve their honour’, implying that this may be a more acceptable reason to seek an abortion. The same can be said about provisions that allow for lower sentences when pregnancies are terminated because of risk to physical or mental health, rape, incest, fetal impairment and lack of support for the child, or where providers are punished more severely when they ‘profit’ from the act. Creating categories of abortion seekers and providers who are considered more legitimate than others risks undermining human rights, limiting access to abortion care and perpetuating discriminatory norms and stereotypes, especially when there is no evidence-based reason for this.^33–38^

## Patterns across countries

This review of penalties for abortion-related offences also illustrates similarities in legal provisions across some countries. It is out of the scope of this paper to interrogate the origin of abortion-related offences in each country for many reasons, including difficulties around translations. However, they may have been influenced by countries’ colonial history.^39 40^ For example, the five countries with an Offences against Persons Act were all once colonised by the UK.^41^ Similarly, the 21 other countries that share a similar provision criminalising abortions have history of being administered or colonised by France.^42^ A better understanding of the origin and historic development of abortion-related offences could shed more light on how some countries amended their colonial abortion-related offences, whether these strategies for change could be used by similarly placed countries, and what other factors—in addition to a shared colonial history—influence commonalities in abortion-related offences across countries.

## Implications for access to quality abortion care

Evidence demonstrates that in countries with restricted access to abortion, rates of unintended pregnancies are higher than in countries where abortion is broadly legal.^43^ Moreover, the incidence of unsafe abortions, one of the leading causes of maternal mortality and morbidity,^44^ is significantly higher in countries with highly restrictive abortion laws.^4^ Marginalised groups are also likely to face more adverse consequences due to restrictive abortions laws.^45 46^ With the adoption of ‘liberal’ or ‘flexible’ abortion laws, reductions in maternal mortality and morbidity have been observed.^31 47^

International human rights law requires countries to undertake measures to reduce maternal mortality and morbidity.^8^ This paper has highlighted the range of penalties for abortion-related offences. However, several barriers to quality abortion care exist in countries and their associated harms have been well documented.^8^ Further research is needed on patterns in abortion-related offences among specific groups of countries, and on how these offences are interpreted in courts. With review and revision (where necessary) of regulatory, law and policy frameworks, countries can continue to work towards a supportive framework of law and policy for access to and provision of quality abortion care.

## Conclusions

Despite evidence of the harms associated with criminalisation, in most countries in the world, abortion seekers, providers and those assisting them may be subject to criminal penalties. The range of possible penalties across countries and associated aggravating and mitigating factors for imposing these penalties, support arguments for the decriminalisation of abortion on the grounds of arbitrariness. These criminal provisions appear to support certain categories of abortion as being more legitimate than others, risking limiting access to abortion care and perpetuating discrimination. Abortions also appear to be regulated differently from other health procedures in most countries. They are predominantly regulated through the criminal law, which may compound the stigma associated with seeking and providing abortions when it is criminalised, with implications for the health and rights of abortion seekers.

## Data Availability

Data are available in a public, open access repository. The data analysed during this study can be accessed in the publicly available Global Abortion Policies Database (http://abortion-policies.srhr.org).
